# On What Could Chinese Mining Enterprises Achieve High-Level Environmental Performance?—Based on the fsQCA Method

**DOI:** 10.3390/ijerph18147290

**Published:** 2021-07-07

**Authors:** Zhengjie Gao, Dayi He, Shuaifang Niu

**Affiliations:** 1 School of Economics and Management, China University of Geosciences, Beijing 100083, China; 2007190071@cugb.edu.cn (Z.G.); 2007200064@cugb.edu.cn (S.N.); 2 Key Laboratory of Carrying Capacity Assessment for Resource and Environment, Ministry of Natural Resources, Beijing 100083, China

**Keywords:** enterprise environmental performance, Chinese mining industry, configurational analysis, fsQCA method

## Abstract

Enterprise environmental performance has causal complexity. The purpose of this paper is to discover the possible combination of conditions for enterprises to achieve high environmental performance. Based on the resource dependence theory, stakeholder theory, and externality theory, this paper constructs the theoretical framework of enterprise environmental performance evaluation and applies the fsQCA method to study the major influencing factors and mechanism of the environmental performance of listed enterprises in the Chinese mining industry. Based on the data from 2016 to 2019, the results show that there are four configurations of multiple factors leading to high environmental performance. Based on these configurations, three possible paths, internally driven, internally–externally driven, and externally driven, are established to improve environmental performance. Further, we also find that, between profitability and government regulation and between enterprise size and board independence are interchangeable condition variables; public attention outweighs other factors for Chinese mining enterprises. Countermeasures and suggestions from perspectives of government supervision, public concern, and enterprise internal governance are proposed at the end the study.

## 1. Introduction

Some adverse effects on the environment have been made by rapid economic development during the past 40 years in China. Fortunately, the government has realized the importance of sustainable development and put the brakes on rapid economic growth. The mining firms are regarded as important sources of environmental pollution, which has become the main object of environmental regulation. In the case of increasingly strict government environmental regulations, environmental performance is becoming more and more crucial to the business activities of mining companies. It is necessary to study the influencing factors of environmental performance and explore how to improve the environmental performance of mining companies, so as to establish a competitive advantage for mining companies in the fierce market competition.

The mining industry is considered to be the culprit in the destruction of the environment and has been given the most stringent control measures [1,2,3]. The impact of the mining industry on the environment is mainly manifested in waste gas, waste water, and solid wastes. In the past decade, the proportion of industrial solid wastes generated by the mining industry has been maintained at about 45%. In 2015, the waste gas emission of mining industry accounted for 1.23% of China’s total emission, the industrial waste water emission accounted for 12%, and the industrial solid wastes accounted for 45.36%. Mining companies have become the main object of environmental supervision. During 2009–2019, the emissions of sulfur dioxide and chemical oxygen demand (COD, an important indicator of water pollution) discharged from the mining industry have shown a downward trend. The proportion of chemical oxygen demand discharged in the mining industry has decreased significantly since 2015, while the proportion of sulfur dioxide emission has decreased steadily in recent years. Thanks to the contribution of the mining industry, China’s environmental performance has been effectively improved in recent years. Therefore, to summarize the influencing factors and paths of the environmental performance of mining enterprises in time is urgent and important for the Chinese government to optimize the environmental regulation policy and continuously improve the environmental performance of mining enterprises. However, there is no consistent conclusion on the influencing factors or mechanisms.

In the research of environmental performance, it is inevitable to involve the financial (economic) performance of enterprises. There are three categories of viewpoints about the relationship between financial and environmental performance [4]. The first viewpoint holds that improving the environmental performance of enterprises will inevitably lead to the increase of costs and the decrease of financial performance, which will not be conducive to the development of enterprises [5,6]. On the contrary, the second viewpoint believes that good environmental performance is the potential competitive advantage of enterprises. Positive environmental behavior or a good corporate image of enterprises can help them to obtain environmental protection license faster than competitors, and enter the new product market earlier. This will be helpful to increase product sales and market share [7]. The third viewpoint reconciles the previous two. It holds that the relationship between financial and environmental performance is an inverted U-shaped type, that is, enterprises with moderate pollution control have the best financial performance [8].

All three viewpoints are verified by empirical research. For example, the first viewpoint was confirmed in [9] by taking the pulp and paper industry as the research object. It is found that environmental management involves a lot of capital expenditure, which will slow down the growth of productivity. Therefore, there is a negative correlation between pollution control and economic development. The second viewpoint was verified by [10,11,12]. The third viewpoint was confirmed by [13,14,15].

In addition to financial performance, environmental performance is also affected by other internal and external factors. In the relevant research, the internal factors mainly include company size, profitability, corporate governance, and other aspects, while the external factors mainly involve government regulation, public concern, and so on.

In terms of company size, it has been shown that enterprises with a larger scale usually have better environmental performance [16,17]. With respect to profitability, there are differences in the selection of proxy indicators, which leads to different research conclusions. ROE are used to measure profitability, and it was found that the profitability of enterprises is positively related to the environmental performance [18,19]. EBITDA (earnings before interest, tax, depreciation, and amortization) and EPS (earnings per share) were used to measure profitability [20], and they found that environmental performance is also positively correlated with profitability. In the aspect of enterprise governance, relevant studies mainly focus on the impact of board independence [21,22], board size [22], and ownership structure [22,23] on corporate environmental performance. Clarkson et al. [21] found that a higher proportion of independent directors is positively related to the environmental performance by the sample of 158 listed companies of Singapore Stock Exchange. Yang [22] took 502 listed companies in China’s heavy pollution industry as samples, and found that the proportion of the largest shareholder has a significant negative correlation with the level of corporate environmental performance; the proportion of independent directors has a positive correlation with the level of environmental performance; the size of the board of directors has no significant correlation with the level of corporate environmental performance. Taking Chinese manufacturing firms as research objects, Liu [23] found that ownership concentration can improve environmental performance significantly. However, the impacts of the shareholding ratio of the largest and second shareholders, total proportion of state-owned shares owned by top 10 shareholders, are not all significantly positive.

There are also various external factors affecting corporation environmental performance. Only a few enterprises will consciously fulfill their environmental responsibilities and pay attention to environmental performance, so government supervision is the most important factor affecting the level of enterprise environmental performance [24]. However, investors, customers, competitors, and the public attention have the most significant impact on environmental performance, while the impact of government regulation is not significant [25]. Li [26] conducted an empirical analysis of 127 firms, and found that environmental regulations, enterprise innovation, and consumers have a positive impact on environmental performance. Zeng [27] found that the government’s assistance to small and medium-sized enterprises with less pollution played a supporting role in improving their environmental performance. Xu [28] found that public attention promoted enterprises to improve environmental performance.

The research methods of enterprise environmental performance are diverse, mainly including regression analysis [17,29,30,31], structural equation model (SEM) [32], game theory modeling [33,34,35,36], system dynamics modeling (SDM) [37,38,39] etc., among which regression analysis is the most common. In general, regression analysis presupposes different influencing factors as independent variables, and takes environmental performance as the only dependent variable, while the SEM method only does not take environmental performance as the only factor variable. Both methods are based on the presupposition of causality. The game analysis method is more applied to the study of the mechanism of government regulation or public supervision on environmental performance. The SDM utilizes the simulation method to study the environmental performance from the macro perspective, which cannot express the micro-factors affecting the environmental performance of enterprises.

From the existing research, it is not difficult to find that there is no consistent conclusion about the influencing factors and mechanisms of enterprise environmental performance. The main reasons for the contradiction are various. First, it is difficult to measure environmental performance. Scholars use different methods to measure environmental performance. Second, most of the existing studies focus on the impact of certain or several factors on environmental performance and ignore the impact of the interaction and joint affection between multiple factors on environmental performance. Hence, these research results have limitations owing to the causal complexity of enterprise environmental performance.

This paper aims to discover possible configurations of factors leading to high environmental performance in Chinese mining industry. Considering the causal complexity of enterprise environmental performance, we try not to separate the influence of other factors when we study one factor. The existing qualitative analysis or correlation analysis does not reveal the comprehensive impact of different combinations of factors on environmental performance, and there is a lack of systematic research on the driving mechanism of environmental performance. As a new sociological research method, fsQCA takes the holistic perspective and configuration thinking, regards the research object as the configuration of different combinations of conditional variables, and finds the set relationship between conditional variables configuration through set analysis, which helps to solve the causal complexity problems such as causal asymmetry and equivalence of multiple schemes [40]. Therefore, based on the resource dependence theory, stakeholder theory, and externality theory, this paper uses the fsQCA method to explore the joint effects of enterprise size, board independence, profitability, government regulation and public attention, and how their interaction affects enterprise environmental performance from a systematic and comprehensive perspective. Thus, it reveals the driving mechanism for Chinese listed mining enterprises to achieve high level of environmental performance.

This paper intends to make three contributions. First, this study introduces the fsQCA method into the research on the influencing factors of enterprise environmental performance, which not only enriches the research methods in this field, but also innovates the epistemological basis of the driving mechanism of enterprise environmental performance, and provides an overall perspective for understanding and explaining the causal complexity of enterprise environmental performance. Second, the conclusion of this paper eases the contradiction in the research of environmental performance to a certain extent. For example, in the existing research, the role of government regulation on enterprise environmental performance is positively correlated [24,26] or not significant [25]. The results show that the relationship between the two depends on the situation of government supervision. In our conclusion, government regulation has a positive effect on environmental performance in configuration 2, but in configuration 1, government regulation has no effect on enterprise environmental performance. Finally, this paper summarizes three paths leading to high environmental performance and provides management enlightenment for listed mining enterprises in China to improve environmental performance.

The paper is organized as follows. Section 2 describes the design of this study, including analytical framework, research method, variable design, case selection, and data calibration. Section 3 presents the main results, followed by some discussion and remarks in Section 4. Section 5 concludes the study and presents some implications and limitations as well.

## 2. Study Design

### 2.1. Theoretical Background

Among the related research on environmental performance, most of them are based on an empirical research framework, using the regression analysis method to analyze the impact of internal and external factors on environmental performance. There is no consistency in choosing the factors affecting the environmental performance. This also shows that the factors affecting environmental performance of enterprises are diverse, and the influence mechanism is complex, so it is difficult to use a single model to answer comprehensively. Therefore, the configuration of various influencing factors is the possible explanation for the difference in enterprise environmental performance and the diverse conclusions about environmental performance research. Qualitative comparative analysis (QCA) is a set theory configuration analysis method based on Boolean algebra. By examining the sufficient and necessary subset relationship between antecedent conditions and results, it can explore how complex social problems are caused by multiple concurrent causalities [41].

**Figure 1:**
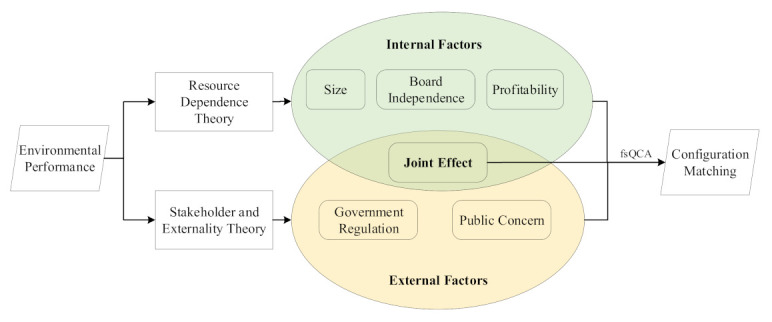
Analytical framework of the study.

Based on the resource dependence theory (RDT) [42], stakeholder theory [43], and externality theory [44], combined with the frequently used variables in related research, this paper constructs the theoretical framework as shown in Figure 1.

According to the RDT, organizations can construct organizational behavior through external restriction and internal power, which makes the organizational initiative adapt to the environment [45,46]. To respond to the external pressure and adapting to the environment, the organization pursues the maximum benefit through internal structure adjustment and optimization. The RDT reveals the dependency relationship between organization and environment, and provides a theoretical basis for the analysis of the internal influencing factors of environmental performance. In this paper, three internal factors, namely, enterprise size, board independence, and profitability, were chosen to reflect the internal influence on enterprise environmental performance.

The stakeholder theory is a theory of organizational management and business ethics that accounts for multiple constituencies impacted by business entities such as employees, suppliers, local communities, creditors, and others [47,48]. According to stakeholder theory, a company is a series of contracts formed with various stakeholders. It is the result of negotiation and transaction between various stakeholders. Investors, managers, employees, customers, suppliers, government departments, communities, and so on, all of them make a specific investment in the company and bear the risks. Therefore, in order to ensure sustainable development, firms should be responsible for both internal and external stakeholders. The externality is a cost or benefit of an economic activity experienced by an unrelated third party [49]. Externality often leads to market and government failure, which provides a theoretical foundation for government intervention in enterprise management. Therefore, in the study of enterprise environmental performance, we must consider the impact of externality. In this paper, government regulation and public concern are chosen to reflect the external influence on enterprise environmental performance.

### 2.2. Methodology

To investigate the factors influencing the environmental performance of Chinese mining enterprises, this paper adopts the fuzzy-set quantitative comparative analysis (fsQCA) method. The software used in this paper is fsQCA 3.0.

QCA is a research method based on Boolean algebra and set theory. It focuses on analyzing how the interaction between condition variables (*X*) affects result variables (*Y*). QCA divides variables into 1 and 0, which are represented by upper and lower case letters, respectively. At the same time, it uses “∼’’ to represent logical NOT, “*” to represent logical AND, “+” to represent logical OR, “→’’to represent cause. For example, a*B*C→D indicates the absence of A and the presence of B and C leads to high level of D. In QCA, we assume that there are *n* conditional variables, then there are $2^{n}$ logical combinations including all conditional variables. These logical combinations can be regarded as the potential configurations that lead to the results. Based on the dichotomy of variables, the truth table is constructed, and the Boolean simplification operation is carried out through simple and difficult counterfactual analysis to obtain the effect result of the combination of conditional variables on the outcome variables. In short, the QCA method aims to find the combination of antecedent conditions that lead to the occurrence or non-occurrence of an outcome [50]. These logical combinations can be regarded as the potential configurations that lead to the results, and the most explanatory conditional configurations can be evaluated by consistency and coverage [51]. The critical standard of consistency is 0.85 [52] or 0.8 [53]. Usually, 0.8 is the lowest standard in most studies. Different from consistency, there is no acceptable minimum threshold for coverage [54].

In practical research, most of the data are continuous, and some important information will be lost if the data are simply dichotomized. Therefore, Ragin introduced fsQCA, which is an extension of QCA. In fsQCA, continuous data need to be converted into membership degree of 0–1. The closer to 1, the higher the degree of belonging to the corresponding set. fsQCA derives the configurations of complex causality involving different conditions with small and medium-sized samples [55], which cannot be analyzed under the classical quantitative methodologies [56,57]. Each case of the sample is explained through a complex combination of different conditions by applying the principles of Boolean algebra and fuzzy algebra [58]. Together with the concept of causality, the outcome can be explained through the various causal configurations [58]. In this way, we can determine the necessity and sufficiency of the combination of conditions to form the outcome [57]. The calculation formulas of consistency and coverage are as follows:

(1) $$\mathrm{Consistency}\left(X_{i}\leq Y_{i}\right)=\frac{\sum \min(X_{i},Y_{i})}{\sum X_{i}}$$

(2) $$\mathrm{Coverage}\left(X_{i}\leq Y_{i}\right)=\frac{\sum \min(X_{i},Y_{i})}{\sum Y_{i}}$$

In the formula, $X_{i}$ is the membership of case *i* in configuration set *X*, and $Y_{i}$ is the membership of case in outcome *Y*. The value range of Consistency ($X_{i}\leq Y_{i}$) is 0–1, and Consistency is an indicator of whether the conditional configuration constitutes a subset of the outcome, which is generally not less than 0.8. The value range of Coverage ($X_{i}\leq Y_{i}$) is also 0–1. Coverage represents the interpretation degree of outcome *Y* caused by combination of *X* [59].

**Figure 2:**
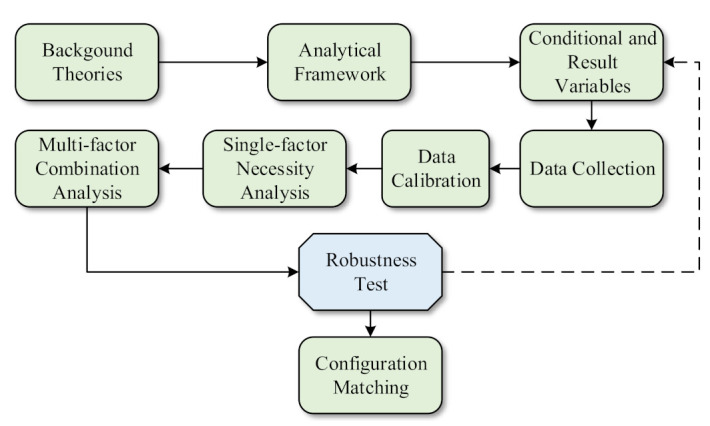
Process of fsQCA method.

The process of fsQCA method can be demonstrated briefly as Figure 2.

Before the implementation of fsQCA, it is necessary to convert the condition value into a fuzzy value, which is called the calibration process. The calibration process is subjective, so it is usually based on researchers’ prior knowledge or results in relevant research to ensure the credibility of research results [60]. Direct calibration, proposed by Ragin [61], is the most frequently used calibration method. In the direct method, a logistic function is utilized to fit the raw data between three qualitative thresholds [57], which define whether the condition belongs to the set [62]. These three thresholds are: (i) full membership (value 1): the cut-off value from which (and above it) the case is in the set; (ii) full non-membership (value 0): the cut-off value from which (and below it) the case is out of the set; and (iii) the cross-over point (value 0.5): this represents the value with greater ambiguity about whether a condition is in or out of the set [61].

### 2.3. Variable Design

In this study, enterprise size (SIZE), enterprise profitability (EP), board independence (BI), government regulation (GR), and public attention (PA) are set to be condition variables. Corporate environmental performance (CEP) is set to be the result variable.

SIZE: Investment in environmental governance has different effects on enterprises of different scales. The larger the scale of enterprises, the lower the unit cost of environmental investment and the less pressure on enterprises. Usually, the natural logarithm of the total assets of an enterprise at the end of the year is used to measure enterprise size [16,17].

EP: Profitability is an important standard to measure the operating level of listed companies, and also an important factor affecting the environmental performance of enterprises. Firms with good profitability provide an economic basis for their environmental governance, and their sufficient funds are conducive to carrying out various environmental governance activities. In the case of poor business performance, firms will not sacrifice business performance for the sake of environmental performance, so the improvement of enterprise environmental performance should be based on good profitability. Referring to the relevant research [18,19], it is usually a comprehensive index to measure the profitability of enterprises by the return on net assets. Therefore, this paper uses the annual return on equity to express the profitability of an enterprise.

BI: Independent directors mainly play a supervisory role in enterprise governance [21,22]. When the business complexity of listed firms rises, more independent directors are needed to supervise the management to ensure scientific and reasonable decision-making. With the increasing pressure of environmental regulation and public attention, enterprises will pay more attention to the investment in environmental management. However, environmental investment has a high risk and long return cycle. Therefore, the enterprise needs to make greater efforts, and the supervision of independent directors is particularly important. In this paper, the proportion of the number of independent directors to the number of the board of directors is used to express the independence of the board of directors.

GR: The government usually supervises the environmental governance of companies by employing administrative supervision, economic reward/subsidiary, or administrative punishment. The authority of the government makes government supervision become the main pressure source on enterprise environmental governance. Government regulation is a kind of direct pressure and rigid constraint, which has a significant impact on the environmental performance of enterprises [24]. In this paper, the annual 120 city Pollution Information Transparency Index (PITI) is used as the proxy variable of government regulation. PITI, developed by the Institute of Public and Environmental Affairs (IPE) and the Natural Resources Defense Council (NRDC), is one of the most comprehensive and objective evaluations of the disclosure of government pollution source regulatory information in China. PITI is a comprehensive score of government supervision on environmental supervision information disclosure, self-monitoring, interactive response, enterprise emission data disclosure, and environmental impact assessment information. The full score is 100. The higher the score is, the greater the degree of government supervision on pollution sources is, and the higher the degree of government supervision on enterprise environment performance is.

PA: With the development of the Internet, public attention is also becoming an important factor affecting the environmental performance of listed companies. Once the listed enterprises have an environmental pollution problem, the real-time reports of the media and the forwarding of netizens will make it easier to expose the pollution problem, and the public attention will form a huge pressure from public opinion. Once the listed companies are reported negatively, their image and reputation will be affected, and this impact is long-term and difficult to recover from [26,27]. In this paper, the annual Baidu search index of the sample enterprise stock code or abbreviation is used to express the degree of public concern.

CEP: Because there is no environmental performance database and recognized environmental performance evaluation system in China, the measurement of environmental performance is not unified. At present, the measurement of environmental performance mainly includes the content analysis method, assignment method, and variable substitution method. Considering the subjectivity of the content analysis method and assignment method and the availability of data, this paper uses the ESG (Environmental, Social, Governance) evaluation to measure the environmental performance of enterprises, which is a kind of enterprise evaluation standard focusing on enterprise environment, society and governance performance [63,64]. ESG gives nine grades from AAA to C. Accordingly, we assign the value from 9 to 1 as the environmental performance measurement of enterprises.

### 2.4. Case Selection

This study focuses on the influencing factors and their configurations of the environmental performance of Chinese mining enterprises. Therefore, the sample is mining enterprises listed on Shanghai and Shenzhen Stock Exchange, and then excluding ST and *ST enterprises, as well as enterprises with missing data; a total of 35 sample enterprises are obtained. This number is in line with the QCA sample number selection rules, that is, the sample number should be greater than $2^{n}$, where *n* is the number of condition variables.

Because the QCA method is only applicable to cross-section data analysis and to avoid some samples from abnormal values, the time effect is neglected in the original QCA method. In recent years, the QCA method has attracted more attention on how to consider time series effect [65]. However, these attempts are still in the stage of theoretical exploration and has not yet been applied specifically. Hence, we did not adopt the TQCA method because of its immaturity.

In this paper, we adopt a compromise approach [66,67,68], which extended the sample scope to three years of data. The average value of the 2016–2018 data is used to represent the conditional variables. Meanwhile, considering the lag of environmental performance [69] and the reliability of data, the result variables are represented by the average value of 2017–2019.

The data sources of this study include enterprise annual report, WIND database, PITI report, ESG, and the Baidu Index official website.

### 2.5. Data Calibration

All the conditions were calibrated to define the degrees of membership through the direct calibration method. For SIZE and BI, this paper takes 95%, 50%, and 5% quantiles as the threshold of full membership, intersection and non-membership, respectively. For ROE, 95% and 50% quantiles are taken as the full membership threshold and intersection, respectively, and the annual deposit rate of the central bank in 2018 is taken as the full non-membership threshold. The crossover threshold of PA is 50%, the threshold of non-membership is 5%, and the threshold of complete membership is set to 1000 referring to the existing research [70]. In order to avoid the loss of sample cases due to half-membership, the threshold of each crossover point is fine-tuned. For GR, the PITI index is a 100-point system, of which 70% are required by laws and regulations for information disclosure, and 30% are beyond the requirements of laws and regulations. In other words, when the PITI score exceeds 70, it has exceeded the information disclosure required by the current laws and regulations. Therefore, the threshold value of full membership is set to 70, the threshold value of crossover is set to 60, and the threshold value of non-membership is set to 40. For CEP, ESG value has 9 grades from AAA to C, which are assigned value from 9 to 1 in this paper. Based on the sample data, the median value is 7. Hence, considering the external standard and rationality of calibration, the three thresholds of CEP are set as 8, 6.9, and 4 respectively. The rules are listed in Table 1.

## 3. Results

### 3.1. Single-Factor Necessity Analysis

In fsQCA, it is necessary to execute single factor necessity analysis before multi-factor combination analysis [57]. In general, if the necessary consistency of a condition is greater than 0.9, then the factor is a necessary condition, and will be excluded in the subsequent analysis [57].

Coverage and consistency are used to determine whether the antecedent conditional configuration is a necessary and sufficient condition for the explained variable [52]. Simply speaking, consistency is similar to the correlation coefficient of regression analysis, and the coverage coefficient represents the proportion of coverage results of a particular solution, that is, the degree of fit of the data results. Coverage can be further divided into raw coverage and unique coverage. Raw coverage indicates the part of the result that is interpreted by a particular configuration, while unique coverage refers to coverage that is independently interpreted by the configuration and does not overlap with other configurations of the same interpreted result [71].

Table 2 shows the single-factor necessity for high environmental performance. It can be found from Table 2 that the consistency level of all conditions is not more than 0.9, so all conditions do not constitute the necessary conditions for high environmental performance.

### 3.2. Multi-Factor Combination Analysis

Configuration analysis studies the sufficiency of the results caused by different configurations with different antecedents. From the perspective of set theory, it is to analyze whether different configuration sets constitute a subset of result sets. The index to determine whether the conditional configuration constitutes the subset of the result is consistent. Some studies point out that the consistency level is not less than 0.75, but most studies take the consistency level not less than 0.8. In this paper, 0.8 is used as the consistency threshold. For the selection of case frequency, the small sample is generally 1, and the large sample should be greater than 1. However, the setting of the case frequency threshold should ensure that at least 75% of the observed cases are included [40]. In this paper, the total number of sample cases is 35, so the frequency threshold is set to 1.

Based on the output of the fsQCA 3.0, four configurations affecting enterprise environmental performance are listed in Table 3.

In Table 3, the consistency value of every configurations and the overall solution consistency are higher than the consistency threshold value (0.8). The overall solution consistency is 0.885, which implies that the four configurations have good explanatory power for high CEP. The overall solution coverage is 0.421, which shows that 42.1% of samples can be classified into the four configurations.

Configuration 1 (SIZE*BI*EP*PA) has a consistency of 0.914 and a raw coverage of 0.290. In this configuration, BI, EP, and PA are core elements to achieve high CEP, and SIZE is the peripheral element. This configuration shows that enterprises with larger size, higher board independence, higher profitability, and higher public attention will have high environmental performance.

In configuration 2 (SIZE*BI*GR*PA), BI, GR, and PA are core elements, and SIZE is the peripheral element. This configuration has a consistency of 0.927 and a raw coverage of 0.269. This configuration shows that enterprises with larger size, higher board independence, stricter governmental regulation, and higher public attention will have high environmental performance.

Configuration 3a (SIZE*EP*GR*PA) and 3b (BI*EP*GR*PA) have the same core elements (EP, GR, and PA), but have different peripheral element (SIZE in 3a, BI in 3b). Configuration 3a has a consistency of 0.913 and a raw coverage of 0.247. This configuration shows that enterprises with larger size, higher profitability, stricter governmental regulation, and higher public attention will have higher environmental performance. Configuration 3b has a consistency of 0.957 and a raw coverage of 0.210. This configuration shows that enterprises with higher board independence, higher profitability, stricter governmental regulation, and higher public attention will have higher environmental performance.

In addition, Table 3 also shows that PA exists in all configurations. Hence, public attention is crucial for a mining enterprise to achieve high environmental performance.

### 3.3. Robustness Test

Two methods are adopted to ensure the credibility of the above results.

First, we test the robustness by increasing the consistency threshold from 0.8 to 0.85 [54,72], it is found that the above results have not changed, which indicates that the results have good robustness.

Second, we re-conduct configuration analysis by adjusting thresholds of CEP in consideration of more strict requirements. The threshold value of full membership is increased from 8 to 9, the threshold value of crossover is changed from 6.9 to 6.5, and the threshold value of non-membership is decreased from 4 to 3.

The results of robustness test is shown in Table 4.

In Table 4, we still obtain four configurations, and they are consistent with the results in Table 3. The consistency, coverage of each configuration, the overall consistency and coverage of the global solution are slightly changed. The overall consistency, and coverage are improved to 0.913 and 0.430, respectively, which are more optimal than they were prior. Therefore, it shows that the results of this study have good robustness.

## 4. Discussion

### 4.1. Three Paths for Mining Enterprises to Achieve High Environmental Performance

**Figure 3:**
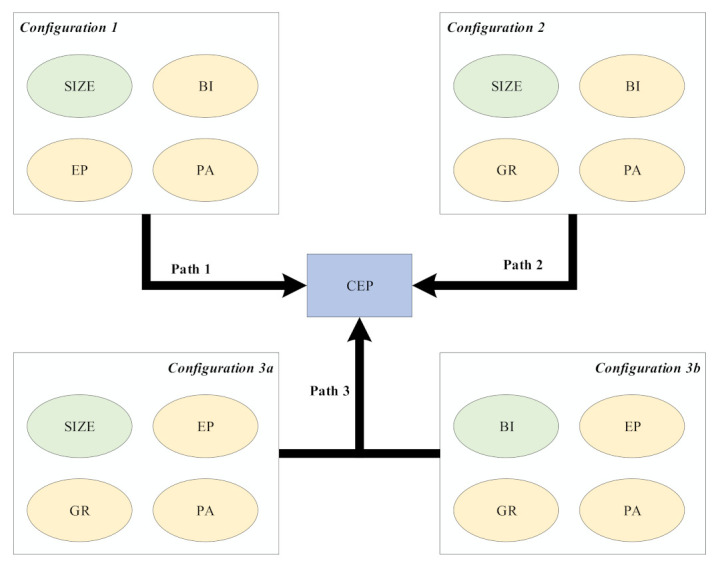
Paths for mining enterprises to achieve high environmental performance. Notes: The green box represents the peripheral elements, and the yellow box represents the core elements to achieve high CEP.

Results generated by fsQCA in Table 3 demonstrate that different combinations of factors can lead to high environmental performance. According to the core conditions contained in the four configurations, Figure 3 summarizes them into three paths to achieve high environmental performance, namely, internally driven (configuration 1), internally–externally driven (configuration 2), and externally driven (configuration 3a and 3b).

The internally driven path corresponds to configuration 1. In this path, two internal factors (board independence and profitability) play a leading role in achieving high environmental performance. Independent directors play an important role in supervision in the decision-making of environmental issues, which can reduce the impact of enterprises on the environment to some extent. Enterprises with strong profitability have sufficient funds to provide funds for environmental governance, and also have the ability to purchase environmental protection equipment to reduce emissions and to control the impact of enterprises on the environment effectively. In addition, in this path, the pressure of public attention also has a positive effect on the improvement of environmental performance. Therefore, under the promotion of these two internal factors, combined with the influence of public attention, constitute the path for mining enterprises to achieve high environmental performance.

The internally–externally driven path corresponds to configuration 2. In this path, the internal promotion factor is the independence of the board of directors, and the external factors are government supervision and public attention. Most of the independent directors of listed mining enterprises are highly educated. Their environmental awareness is conducive to the enterprise to achieve good environmental performance. As one of China’s heavy polluting industries, mining industry is often the focus of government environmental regulation. In recent years, with China’s increasing emphasis on environmental governance, government regulation has become an important factor affecting corporate environmental governance. In response to government regulation, enterprises can only reduce the impact of their business activities on the environment, while increasing investment in environmental governance. Therefore, government regulation has become an important external factor for enterprises to improve their environmental performance. In addition, due to the highly developed Internet, once the listed enterprises have a major accident endangering the environment, the network public opinion is easy to make the involved enterprises fall into the crisis of public relations. Therefore, public attention is also an important external factor in this path. In this path, the linkage of internal and external factors makes mining enterprises achieve high environmental performance.

The externally driven path corresponds to configuration 3a and configuration 3b. In this path, the external pressure is the government’s supervision and the public’s attention, and the internal factor is the profitability or the independence of directors. On the issue of environmental management, the government’s supervision means for enterprises are mainly to formulate and improve environmental protection laws and regulations, pollutant emission supervision, administrative punishment, and reward, which is a kind of constraint for enterprises. On the one hand, the popularity of the Internet means the environmental problems of enterprises are quickly exposed, the reputation of enterprises will be affected, and this impact is long-term and difficult to recover from, which will form a huge pressure on enterprises; on the other hand, with the continuous improvement of public awareness of environmental protection, enterprises will pay more and more attention to the public’s feelings in the process of environmental management. Therefore, driven by these two external pressures, it constitutes another path for mining enterprises to achieve high environmental performance.

### 4.2. Relationship between Conditions

One advantage of configuration analysis is to identify the interaction between conditions.

It can be seen from configuration 1 and configuration 2 that there is an obvious substitution relationship between profitability and government regulation. The combination of larger SIZE, high BI and high PA, strong EP, or strong GR can lead to high environmental performance. This shows that the three factors of SIZE, BI, and PA only need to be combined with strong EP or GR to promote environmental performance, without the existence of both conditions.

In configuration 3a and configuration 3b, there is an obvious substitution relationship between enterprise size and board independence. Strong EP, strong GR, and high PA can promote environmental performance only by combining with any of the two conditions of SIZE or BI.

Finally, public attention as a core condition exists in all configurations. This shows that public attention is most important for enterprises to achieve good environmental performance. Profitability and government regulation as the core conditions appear three times in the four configurations, which shows that they are more important for enterprises to achieve good environmental performance.

## 5. Conclusions and Implications

Enterprise environmental performance is affected by internal and external factors. These influencing factors are diverse, and the influence effect and mechanism are various. Therefore, this paper studies the main factors that affect the environmental performance of enterprises and the possible configuration for high environmental performance. Based on the data of Chinese listed mining companies from 2016 to 2019, using the fsQCA method, this paper obtains the following main findings.

First, the high environmental performance of China’s mining listed enterprises is the result of multiple factors. Any single factor can neither be the necessary condition nor the sufficient condition for high environmental performance. This paper finds that there are four configurations for Chinese mining enterprises to achieve high environmental performance, and each configuration is composed of multiple factors.

Second, there are three paths for Chinese mining enterprises to achieve high environmental performance: internally driven, internally–externally driven, and externally driven. The internally driven path is mainly the matching between the independence of the board of directors and profitability to drive mining enterprises to obtain high environmental performance. The internally–externally driven path is the synergy of the independence of the board of directors, government supervision, and high public concern to make mining enterprises obtain high environmental performance. The externally driven path indicates that the pressure effect of government regulation and public attention can also make mining enterprises achieve high environmental performance.

Third, in the process of achieving a high environmental performance of Chinese mining enterprises, there is an alternative relationship between profitability and government regulation, and there is an alternative relationship between enterprise size and independence of the board of directors. This means that it is beneficial for Chinese mining listed companies to allocate relevant resources and optimize their capabilities based on their conditions, so as to achieve high environmental performance. For example, for enterprises with strong profitability, high government supervision, and public concern, high environmental performance can be achieved by expanding the scale of enterprises, or by improving the independence of the board of directors.

Fianlly, in the process of pursuing a high environmental performance of Chinese mining enterprises, public attention is very important to improve their environmental performance, and the role of profitability and government supervision cannot be ignored.

Based on the above conclusions, this paper puts forward the following suggestions for government regulation and enterprise environmental management.

First, the government should actively improve the system of environmental laws and regulations, establish and improve standardized, unified, specific, and operable environmental protection regulations and guidelines, and ensure the implementation of environmental protection policies, so as to play the regulatory role in the governance better.

Second, the public should strengthen the publicity of environmental protection to make the awareness of protecting the environment deeply rooted in the hearts of the people. Further, a network monitoring and reporting platform of environmental pollution for listed enterprises should be developed to give full play to the power of the network and motivate the enthusiasm of the public to participate in environmental governance.

Third, listed mining enterprises should formulate the development strategy from a long-term perspective, change passive governance to active avoidance in environmental protection. The enterprise should strengthen internal governance, actively fulfill social responsibilities, entitle independent directors with sufficient power in environmental pollution supervision. Mining enterprises should focus on improving the profitability of enterprises to provide a financial basis for improving the environmental performance of enterprises.

The endeavors of this paper was to study the factors and paths that affect the environmental performance of enterprises. Taking Chinese mining enterprises as samples and by the fsQCA method, we achieve four possible configurations and three paths for mining enterprises to achieve high environmental performance. It might be of significance in the research of enterprise environmental performance. However, it also should be noted that this research has limitations. First, for the internal influencing factors of environmental performance, this paper only selects three variables. Due to the complexity of environmental performance, there may be other internal influencing factors. Second, since QCA is limited by cross-section data, this paper investigated the environmental performance from a static perspective. In future research, time effects can be taken into consideration.

## Figures and Tables

**Table 1:** Rules of calibration.

Variable	Full-Membership Threshold	Crossover Point	Non-Membership Threshold
SIZE	25.994	23.430	20.982
BI	0.430	0.355	0.333
EP	0.180	0.066	0.015
GR	70	60	40
PA	1000	600	314.867
CEP	8	6.9	4

**Table 2:** Necessity analysis of single factor for high CEP.

Condition Variables	High CEP	
	Consistency	Coverage
SIZE	0.729	0.775
∼SIZE	0.487	0.542
BI	0.569	0.716
∼ BI	0.623	0.595
GR	0.562	0.737
∼GR	0.576	0.534
EP	0.566	0.672
∼EP	0.654	0.655
PA	0.721	0.711
∼PA	0.442	0.535

**Table 3:** Multi-factor combination path analysis of high CEP.

Configuration	Solution			
	1	2	3a	3b
SIZE	•	•	•	
BI	●	●		•
EP	●		●	●
GR		●	●	●
PA	●	●	●	●
consistency	0.914	0.927	0.913	0.957
raw coverage	0.290	0.269	0.247	0.210
unique coverage	0.091	0.070	0.049	0.012
overall solution consistency	0.885			
overall solution coverage	0.421			

Note: • indicates that the condition variable exists. The large circle represents the core element, and the small circle represents the peripheral element. “Blank” indicates that the condition variable may or may not appear in the path.

**Table 4:** Multi-factor combination path analysis of high CEP.

Configuration	Solution			
	1	2	3a	3b
SIZE	•	•	•	
BI	●	●		•
EP	●		●	●
GR		●	●	●
PA	●	●	●	●
consistency	0.945	0.942	0.951	0.976
raw coverage	0.295	0.269	0.254	0.211
unique coverage	0.096	0.069	0.054	0.011
overall solution consistency	0.913			
overall solution coverage	0.430			
